# Postoperative Arrhythmias after Cardiac Surgery: Incidence, Risk Factors, and Therapeutic Management

**DOI:** 10.1155/2014/615987

**Published:** 2014-01-06

**Authors:** Giovanni Peretto, Alessandro Durante, Luca Rosario Limite, Domenico Cianflone

**Affiliations:** IRCCS Ospedale San Raffaele, Università Vita-Salute San Raffaele, Via Olgettina 60, 20132 Milan, Italy

## Abstract

Arrhythmias are a known complication after cardiac surgery and represent a major cause of morbidity, increased length of hospital stay, and economic costs. However, little is known about incidence, risk factors, and treatment of early postoperative arrhythmias. Both tachyarrhythmias and bradyarrhythmias can present in the postoperative period. In this setting, atrial fibrillation is the most common heart rhythm disorder. Postoperative atrial fibrillation is often self-limiting, but it may require anticoagulation therapy and either a rate or rhythm control strategy. However, ventricular arrhythmias and conduction disturbances can also occur. Sustained ventricular arrhythmias in the recovery period after cardiac surgery may warrant acute treatment and long-term preventive strategy in the absence of reversible causes. Transient bradyarrhythmias may be managed with temporary pacing wires placed at surgery, but significant and persistent atrioventricular block or sinus node dysfunction can occur with the need for permanent pacing. We provide a complete and updated review about mechanisms, risk factors, and treatment strategies for the main postoperative arrhythmias.

## 1. Introduction

Arrhythmias are very common complications after cardiac surgery and represent a major source of morbidity and mortality. Atrial tachyarrhythmia are the most common postoperative heart rhythm disorder. Ventricular arrhythmias and bradyarrhythmias are less frequent [1].

The clinical significance of each arrhythmia depends upon its duration, ventricular response rate, underlying cardiac function, and comorbidities. In fact, arrhythmias that may be well tolerated in a younger patient can be a major cause of morbidity and mortality after cardiac surgery for congenital heart disease [1, 2].

Arrhythmia management includes correction of transient and correctable predisposing factors, as well as specific therapy for the arrhythmia itself. The urgency for and type of the required treatment are determined by the clinical presentation of the arrhythmia. Self-terminating arrhythmias, in the setting of a transient stress and without overt cardiac disease, often need no therapy at all. On the other hand, the development of hemodynamically significant arrhythmias in patients under critical stress conditions like systemic infections or persistent pericardial effusion need a therapy for restoring a stable clinical status.

## 2. Pathophysiology

Many perioperative factors have been implicated in atrial and ventricular susceptibility to postoperative arrhythmias (POAs), but their relative role is still uncertain. Risk factors can be classified into patient and surgery related.

### 2.1. Patient-Related Risk Factors

Various patient-related clinical and nonclinical risk factors for postoperative arrhythmias have been described.

*(i) Age*. Increasing age has been demonstrated to be correlated with the development of POAs. In fact age-related structural or electrophysiologic changes appear to lower the threshold for postoperative atrial tachyarrhythmias in the elderly [2–4]. In a review of 915 consecutive adults in sinus rhythm who underwent valve surgery, the odds ratio for developing postoperative atrial fibrillation (POAF) was 1.51 per decade [5]. 

*(ii) Structural Heart Disease*. Postoperative dysrhythmias are most likely to occur in patients with structural heart disease. Patients undergoing cardiac surgery often have the substrate of atrial enlargement or elevation in atrial pressures. These changes predispose to atrial tachyarrhythmias. The propagation of reentrant circuits during atrial fibrillation (AFib) can be promoted by larger atrial sizes that can support multiple circuits. Similarly, in patients with cardiomegaly, underlying structural heart disease can play as a ventricular arrhythmogenic substrate. Previous history of arrhythmias (particularly AFib), cardiac surgery and POAs, severe right coronary artery stenosis [6], sinus nodal or atrioventricularnodal artery disease [7–9], and mitral valvular disease (particularly rheumatic mitral stenosis) represents other important risk factors. Also preoperative brain natriuretic peptide plasma concentration is a predictor of POAs [10]. 

*(iii) Extracardiac Comorbidities*. Reported extracardiac risk factors for postoperative arrhythmias include obesity [11], previous stroke, and history of chronic obstructive pulmonary disease [12].

### 2.2. Surgery-Related Risk Factors

*(i) Trauma and Inflammation*. The trauma of cardiac surgery predisposes patients to atrial and ventricular arrhythmias. Inflammatory mechanisms have been proposed in the development of POAF since its incidence peaks at postoperative day 2-3. Inflammation is often related to the development of clinically-evident or silent pericarditis. Unfortunately clinical criteria, such as pericardial rubs, electrocardiogram changes, fever, and pleuritic chest pain, correlate poorly with postoperative pericarditis and supraventricular arrhythmias. However one study reported that 63% of patients with pericardial effusions had supraventricular arrhythmias, compared with 11% of patients without effusions [13]. 

*(ii) Hemodynamic Stress*. Atrial changes occurring at the time of surgery, such as acute atrial enlargement, ischemia, hypertension, and trauma from cannulation, are known risk factors for postoperative atrial arrhythmias. Postoperative pleural effusion requiring thoracotomy and postoperative pulmonary edema have also been described as possible risk factors [14]. Hemodynamic changes can also trigger focal arrhythmias. In fact atrial stretch, hypertension, pressure and volume shifts, and heightened catecholamine states can trigger AFib foci from the pulmonary veins [15]. 

*(iii) Ischemic Injury*. Atrial and ventricular ischemia and/or infarction may play a role as a trigger for POAs. Hypoxemia, hypercarbia, endogenous or exogenous catecholamines, acid-base imbalances, and drug effects, as well as mechanical factors such as instrumentation, are well-recognized causes of myocardial focal ischemia. This may trigger a POA [16]. Cardiopulmonary bypass and cross-clamp times, type of cardioplegia, and coronary artery bypass graft (CABG) surgical technique are also critical in determining ischemic injury. The incidence of AFib has been demonstrated to be lower after off-pump CABG than conventional CABG. Off-pump CABG is also associated with a lesser degree of inflammation [17]. 

*(iv) Perioperative Drugs*. Preoperative use of beta adrenergic blockers [18, 19] and digoxin [20] has been studied. Beta blocker withdrawal has been associated with an increased rate of postoperative supraventricular arrhythmias [21]. A state of heightened catecholamine effect occurs because chronic beta blocker use leads to a higher density of beta-adrenergic receptors. Preoperative digoxin use has been described as a risk factor in some [3, 22] but not all studies [23]. Inotropic agents are known to increase sinoatrial node automaticity and decrease AV nodal conduction time. In humans, dobutamine has been reported to induce ventricular ectopic activity in 3% to 15% of patients [24]. Controversial results have been published for dopamine, which is more likely to be associated with dose-related sinus tachycardia or AFib [25]. Finally, short-term intravenous administration of the phosphodiesterase inhibitors amrinone and milrinone has been reported to cause VPB and short runs of VT in up to 17% of patients [24]. 

*(v) Electrolytes Disorders*. Hypokalemia may provoke postoperative arrhythmias through the well-known alteration of the electrophysiologic properties of cardiac myocytes, including an increase in phase 3 depolarization, enhanced automaticity, and decreased conduction velocity [26]. This might be particularly evident in the atria, where changes in inward-rectifier potassium currents are supposed to act as profibrillatory mechanisms [26]. However, hypokalemia is more often associated with ventricular tachyarrhythmias [27]. Moreover, it is worth noting that arrhythmogenesis is often multifactorial. The attribution of an arrhythmia to a single predisposing factor may oversimplify a complex situation, for example, hypokalemia predisposes to the development of perioperative ventricular arrhythmias. Catecholamine release, however, increases cellular potassium uptake and thus decreases serum potassium levels [27]. The role of magnesium remains controversial. Magnesium levels frequently decrease after cardiac surgery and low serum levels correlate with an increased incidence of POAs. However magnesium supplementation has produced conflicting results. Magnesium supplementation should aim to achieve adequate magnesium blood levels [28, 29]. 

*(vi) Preservation of Anterior Fat Pad*. Fat pads enclosing the surface of the heart have been demonstrated to contain parasympathetic ganglia [30]. The anterior fat pad is routinely dissected and often completely removed during cardiac surgery, because it is located where the aortic cross-clamp is typically placed. It has been suggested that preserving the anterior epicardial fat pad during cardiac surgery may reduce the risk of POAs, in particular AFib [31, 32]. 

*(vii) Special Conditions*. POAs are known complications after open heart surgery for congenital heart disease [33–35] and after heart transplant. Specific factors that may cause or increase the risk of POAs in the setting of congenital heart diseases are preexisting myocardial compromise by the cardiac defect and complex operation with extensive scars and suture lines [36]. As for the transplanted heart, several mechanisms for arrhythmogenesis have been described as well: ischemia during organ preservation, wide surgical suture lines, acute and chronic rejection, accelerated atherosclerosis and denervation may contribute to the formation of the arrhythmogenic substrate [37–42].

Despite these associations, none of these risk factors have adequate predictive accuracy to identify patients who will develop POAs. As a result, risk models have been created based upon several variables, in particular for atrial tachyarrhythmias [2, 43].

## 3. Supraventricular Tachyarrhythmias

AFib is the most common arrhythmia after cardiac surgery, and it is often associated with other atrial tachyarrhythmias, such as atrial flutter (AFlu), premature atrial complexes, and multifocal atrial tachycardia.

### 3.1. Epidemiology

AFib has been reported in up to 15 to 40% of patients in the early postoperative period after CABG, in 37 to 50% after valve surgery, in as many as 60% undergoing valve replacement plus CABG, and in 11 to 24% after cardiac transplantation [44–46]. The incidence of POAF in patients undergoing CABG consistently increases with older age [3, 47].

Postoperative AFib and AFlu most often occur within the first few days after surgery, with a peak incidence on postoperative days 2 and 3 [4, 48]. Accurate data from a prospective multicenter study of 4,657 patients undergoing surgery [2] reported that the majority of first episodes of AFib occurred by day two, while the majority of recurrent episodes occurred by day three. More than 40% of patients with AFib had more than one episode.

Among patients with POAF but without a prior history of atrial arrhythmias, AFib is usually self-limited as 15 to 30% convert to sinus rhythm within two hours and up to 80% within 24 hours [49, 50]. The mean duration of AFib in one report was 11 to 12 hours and more than 90% of patients were in sinus rhythm six to eight weeks after surgery [50, 51]. In another report only 3 out of 116 patients who developed AFib after CABG were still in AFib at six weeks [52]. The administration of antiarrhythmic drugs did not appear to alter the course.

The relative contributions of reentrant versus focal mechanisms to postoperative atrial arrhythmias development have not been clearly established. Apart from common patient-related and surgery-related risk factors for postoperative arrhythmias, several electrophysiologic parameters may promote the development of AFib, such as dispersion of atrial refractoriness, atrial conduction velocity, and atrial transmembrane potentials [53]. Nonuniform atrial conduction is greatest on postoperative days two and three, and the longest atrial conduction time is greatest on day three [54]. These abnormalities are in keeping with the time of greatest risk for AFib, which has a peak incidence on days two to three [44]. AFib development after CABG has been associated with increased expression and heterogeneity in distribution of connexin-40, an intercellular gap junction protein [55]. These changes could result in differences in resistive and conductive properties of atrial myocardium. Preoperative increase in P wave duration on surface (>116 ms) [56] or on signal-averaged (>140 ms) ECG [57, 58] has been also described as a potential risk factor for atrial tachyarrhythmias.

AFlu is often a late complication of cardiac surgery [59]. AFlu has a reentrant mechanism and may involve atypical isthmuses between natural barriers, atrial incisions, and scar as well as the cavotricuspid isthmus [60, 61].

### 3.2. Clinical Course and Diagnosis

Tachyarrhythmias may decrease diastolic filling and cardiac output and increase myocardial oxygen consumption thus resulting in hypotension and myocardial ischemia. POAF onset is frequently symptomatic because it is often associated with rapid ventricular rates. AFib, through an alteration of normal atrioventricular synchrony, can result in a 15 to 25% reduction in cardiac output [3]. Loss of the atrial “kick” may cause a dramatic increase in pulmonary pressures, especially in patients with diastolic dysfunction [62].

In the majority of cases, the diagnosis of POAF can be confirmed, simply, based on 12-lead ECG findings. In fact, AFib usually presents with an abrupt change in rhythm with loss of P waves on surface ECG or telemetric monitoring [7, 63]. However, the diagnosis of AFib, AFlu, or other forms of supraventricular tachycardia can be confirmed by using atrial electrograms obtained from temporary atrial epicardial pacing wires that are often routinely placed at the time of cardiac surgery [63].

### 3.3. Prognosis

Supraventricular tachyarrhythmias are a common cause of morbidity after cardiac surgery. Although POAF is often self-limiting, symptoms, hemodynamic compromise, and risk of thromboembolism can present. Clinical effects of arrhythmias depend on ventricular rate, ventricular function, and arrhythmia duration [64].

POAF has been associated with an increase in in-hospital stroke [64–66]. In a series of almost 4,000 patients, the incidence of stroke was significantly higher in those who developed AFib (3.3 versus 1.4%) [3]. However underlying comorbidities, such as older age, previous cerebrovascular disease, presence of a carotid bruit, peripheral vascular disease, and cardiopulmonary bypass time, play a key role in the development of in-hospital stroke in adjunction to the arrhythmia itself [67–69]. In fact, in a review of 2,972 patients undergoing CABG and/or valve surgery, POAF did not increase the risk of stroke immediately after surgery and was associated with delayed stroke only if accompanied by a low cardiac output syndrome (3.9 versus 1.9%) [70]. Prophylactic therapy for POAF did not lower the incidence of in-hospital stroke [71].

POAF development is associated with a prolonged length of hospitalization [3, 14, 48]. According to some authors, hospital length of stay after CABG surgery can be extended by 2–4 days with additional costs of several thousand dollars per patient after POAF [48]. However, this effect has become less prominent with current cardiac surgical care [71, 72].

POAF may also identify a subset of patients with increased in-hospital and long-term mortality [2, 64]. This was suggested by a retrospective study of 6,475 patients undergoing CABG at a single institution: nine hundred and ninety-four patients (15%) developed AFib. Patients with AFib had significantly greater in-hospital (7.4% versus 3.4%) and four-year mortality (26% versus 13%) but also more comorbidities (i.e., older age, hypertension, and left ventricular hypertrophy) [64].

### 3.4. Therapy

The treatment of postoperative supraventricular tachyarrhythmias requires a comprehensive management:

- antithrombotic therapy to prevent thromboembolic events;
- antiarrhythmic treatment, including both rate and rhythm control strategies.

*Antithrombotic Therapy*. Patients with AFib are at increased risk of thromboembolic events. Thromboembolic risk is increased in patients with AFib of more than 48 hours duration and is greater in patients with certain high-risk features, such as rheumatic mitral valve disease and previous thromboembolism, hypertension, or heart failure [73]. However, the role of short- and long-term anticoagulation in patients with POAF is not clear. In fact there is uncertainness regarding the reduction of the incidence of stroke on anticoagulants. Moreover the bleeding risk is increased with anticoagulation in the immediate postoperative period. Many patients with new-onset AFib after cardiac surgery spontaneously revert to and maintain sinus rhythm [44, 50, 51]; thus anticoagulation may be unnecessary. However, when anticoagulation is initiated, the patient should be carefully monitored. The 2008 ACCP guidelines on antithrombotic therapy in AFib suggest oral anticoagulation for patients with POAF after cardiac surgery lasting >48 hours if bleeding risk is acceptable [74]. The recommended target INR is 2.5 (range 2.0 to 3.0). Also according to the 2004 ACC/AHA guidelines oral anticoagulation should be continued for four weeks, particularly in patients with risk factors for thromboembolism [75]. Patients with a prior history of AFib or who remain in permanent AFib after surgery should be anticoagulated with warfarin based upon their long-term embolic risk, similarly to other patients with this arrhythmia. The role of the early use of heparin as a bridge to therapeutic oral anticoagulation is even less clear. Although neither the ACC/AHA nor the ACCP guidelines specifically addressed this issue [74, 75], a large retrospective series suggested that the routine use of heparin as a bridge is not necessary in this setting [76].

The risk of embolism of postoperative AFlu is unknown and the need for anticoagulation during AFlu or prior to cardioversion is uncertain. However similarly to POAF the duration of the arrhythmia is generally limited and the risk of embolism is expected to be low. This was illustrated in a report of 122 patients with AFlu after cardiac surgery who underwent external electric cardioversion. None of the patients had embolic events regardless of the anticoagulation status [77]. However, due to the absence of definitive data, we feel that the anticoagulation recommendations for POAF should also be applied to postoperative AFlu.

Finally, new devices are being investigated that occlude the left atrial appendage to try to prevent embolization. Clinical trials showed that patients who received a device had a slightly lower risk of stroke than otherwise seen in clinical practice. However, safety and efficacy are still being determined [78].

Interestingly, to stratify risks and benefits of oral anticoagulation therapy in critically ill patients, a number of scores have been introduced in clinical practice. According to the ESC guidelines [79], before starting an anticoagulation strategy both ischemic and bleeding risks should be assessed, respectively, by the CHA2DS2-VASc [80] and the HAS-BLED [81] score systems. The indication to start an anticoagulation therapy depends, patient by patient, on the balance between individual ischemic and hemorrhagic risk. However, the main limitations to the clinical use of these scores are the following. First, many risk factors (i.e., age, hypertension, and history of stroke) are shared between these scoring systems. Second, continuous variables (i.e., age, liver, and kidney dysfunction) are used in a binomial way (i.e., as yes/no phenomena), resulting in an oversimplification [82].

*Antiarrhythmic Therapy*. In patients with POAF there is a higher threshold for long-term antiarrhythmic therapy. In fact, at least in patients without a prior history of AFib, the arrhythmia reverts spontaneously to sinus rhythm within 24 hours in 80% of cases [49, 50] and by six to eight weeks in over 90% [51, 83]. The therapeutic options for AFib include rate control and electrical or pharmacologic cardioversion [73].

The optimal approach to rate control strategy is uncertain. Rate control strategy with anticoagulation represents a reasonable first approach to hemodynamically stable patients due to the transient nature of the arrhythmia. This approach is associated with early discharge and has been demonstrated to be safe [49, 84]. In addition, rate control seems to be as effective as cardioversion, as illustrated in a prospective, randomized trial [50].

Electrical cardioversion of well tolerated PAOF might be unnecessary due to the self-limited course and the high initial recurrence rate. Cardioversion in asymptomatic patients may be reasonable when well tolerated AFib onset occurs at the time of anticipated hospital discharge or when POAF does not spontaneously terminate within 24 hours.

Cardioversion is indicated for symptomatic patients. Sinus rhythm restoration can be achieved by electric cardioversion of AFib or AFlu or pace-termination of AFlu [85]. Electrical therapy for AFib involves direct current external transthoracic cardioversion. There are three options when the arrhythmia is refractory to transthoracic cardioversion:

- the administration of intravenous antiarrhythmic drug before repeating electrical cardioversion [44];
- internal, low-energy defibrillation with transvenous electrodes or epicardial wires placed during surgery [86, 87];
- a “double defibrillator” technique in which two pairs of orthogonally placed external patch electrodes are discharged simultaneously [88, 89].

Pharmacologic cardioversion can be achieved with class IA, IC, or III antiarrhythmic drugs, either orally or by the intravenous route. Pharmacologic cardioversion of AFib should be considered when reversion is desirable but the patient's status makes anesthesia for electrical conversion potentially difficult. The efficacy of antiarrhythmic drugs for reversion of POAF is similar to that in AFib not related to surgery [90, 91]. A meta-analysis of 91 small studies concluded that the relative efficacy of the most commonly used antiarrhythmic drugs is similar [92]. Class IA (quinidine, procainamide, and disopyramide), class IC (flecainide and propafenone), and class III (amiodarone, sotalol, ibutilide, and dofetilide) drugs were all more effective than placebo, converting 40–60% of patients compared to about 30% with placebo. Comparisons of class IA or class IC versus class III drugs did not show any difference. The efficacy of each single antiarrhythmic agent in the setting of POAF has been described in the literature [93]: quinidine 64%; procainamide 61–87%; disopyramide 48–85%; flecainide 60–86%; propafenone 43–76%; amiodarone 41–93%; sotalol 35–85%; dofetilide 36–44%; and ibutilide 57%. Patients who do not convert to sinus rhythm after a 24-hour trial of drug therapy typically undergo electrical cardioversion.

Beyond pharmacological or electrical cardioversion, other therapeutic strategies apply to AFib management. Among them, radiofrequency ablation is generally tried in patients in whom one or two drugs have failed to control AFib. The efficacy of a single radiofrequency ablation procedure is in the range of 60% to 80% for paroxysmal AFib and 40% to 60% for persistent AFib [94]. However, inconsistent data about results of LA ablation following POAF have been published so far [95].

Finally, in the presence of preoperative AFib, surgical ablation can be performed together with cardiac surgery. The new 2012 HRS/EHRA/ECAS Expert Consensus Statement on catheter and surgical ablation of AFib suggests that all patients with symptomatic AFib undergoing other cardiac surgery should be considered for AFib ablation, provided that the operation is performed by an experienced surgeon [96]. A success rate between 65% and 95% was reported by a number of retrospective studies using a variety of surgical procedures for the treatment of AF with concomitant mitral or other cardiac operations [97, 98].

### 3.5. Recurrence and Prophylaxis

Recurrent AFib after cardiac surgery may require a trial with a different antiarrhythmic drug followed by repeated electrical cardioversion. Alternatively, in the case of well tolerated arrhythmias, the patient can be discharged on oral anticoagulant; a new attempt of cardioversion can be performed after four to six weeks after recovery from surgery. However the persistence of the arrhythmia at hospital discharge is infrequent. The persistence at followup is even less frequent. Even through a rate-control strategy, over 90% of patients are in sinus rhythm 2–4 weeks after onset of the arrhythmia [99]. Achievement and maintenance of sinus rhythm may be more difficult in patients with valvular disease but can often be done with a reasonable expectation of success after valve surgery. However, success may be limited when the duration of AFib is >1–3 years, the left atrial diameter is >5.2 cm, or patients are older [100, 101].

Prophylactic therapy for POAF is routinely used, especially for patients with clinical risk factors [44]. 

*(i) Beta Blockers*. The most widely used prophylactic therapy is the administration of a beta blocker, with or without digitalis [2, 102, 103]. Beta blocker administration reduces the incidence of POAF from 30–40% to 12–16% after CABG alone and from 37–50% to 15–20% after valve surgery [71]. Beta blockers also reduce the ventricular rate when AFib occurs. The 2004 ACC/AHA guidelines update on CABG gave a class I recommendation to preoperative or early postoperative beta blocker therapy in patients without a contraindication [75]. The greatest benefits are seen when beta blockers are initiated prior to or immediately after surgery and are independent of the agent or dose. In the European Society of Cardiothoracic Surgery 2006 guidelines [104] the perioperative use of beta blockers is recommended as the first choice in all patients undergoing cardiac surgery, unless otherwise contraindicated. Intravenous administration is more effective in some series [105] but the tolerance is poor due to the development of side effects [106]. Different beta blockers have been compared. In two small series the incidence of AFib was reduced with carvedilol as compared to metoprolol [107, 108]. Although nonselective beta blockers can cause bronchospasm in patients with chronic obstructive pulmonary disease, beta-1 selective beta blockers (e.g., atenolol or metoprolol) appear to be safe even when there is a bronchospastic component [109]. The optimal duration of beta blockers therapy for prevention of POAF is uncertain, but beta blockers are often continued at least until the first postoperative clinical visit, depending on the underlying heart disease. However, many patients undergoing cardiac surgery have an independent indication for beta blocker therapy (e.g., previous myocardial infarction, heart failure, or hypertension); thus therapy should be continued long term. 

*(ii) Sotalol*. Sotalol is a class III antiarrhythmic agent that also has beta blocking activity. Prophylaxis with sotalol has been shown to protect from AFib after cardiac surgery [110, 111]. Although it has been suggested that sotalol might be more effective than a beta blocker [44, 54], this was not confirmed in a meta-analysis [71]. Sotalol is effective when started 24 to 48 hours before surgery or within four hours after surgery [110, 112]. The 2004 ACC/AHA guidelines update on CABG gave a class IIb recommendation to low-dose sotalol to reduce the incidence of AFib in patients who are not candidates for traditional beta blockers [75]. 

*(iii) Amiodarone*. Amiodarone lowers the incidence of POAF by about 40% to 50% [50, 113, 114]. In the meta-analysis cited above, amiodarone provided similar protection against POAF compared to beta blockers and sotalol (OR 0.54, 95% CI 0.44 to 0.67) [71]. The use of prophylactic amiodarone was assessed in a meta-analysis of ten trials [115]. Amiodarone was associated with a significant reduction in the rate of postoperative AFib or AFlu (22% versus 35%, relative risk 0.64, 95% CI 0.55–0.75). A similar benefit was seen in the PAPABEAR study, the largest randomized trial of amiodarone in cardiac surgery [116]. Several different preoperative regimens of amiodarone have been studied. It has been given orally one to seven days before surgery [56, 57], intravenously immediately after surgery [113], or intravenously for 24 hours followed by oral therapy for four days [59]. Although some authors suggested that amiodarone might be more effective than a beta blocker for the prevention of POAF [117], this was not confirmed in the 2004 meta-analysis [71]. It is worth reminding the rare complication of intravenous amiodarone, the onset of acute respiratory distress syndrome in the postoperative period [118]. The 2004 ACC/AHA guidelines suggested preoperative amiodarone in patients who have a contraindication to beta blockers and are at high risk for POAF [115]. High risk features include previous AFib and mitral valve surgery [44]. 

*(iv) Other Drugs*. Alternative antiarrhythmic drugs, such as digoxin [119–121], calcium channel blockers (CCBs) [122], and class I antiarrhythmic agents [123, 124], do not appear able to prevent the overall incidence of AFib. Also magnesium supplementation should not be recommended as it is probably ineffective [125]. 

*(v) Pleiotropic Agents*. Some nonantiarrhythmic drugs have the potential to protect against the development of perioperative AFib due to their antioxidant or anti-inflammatory properties. Among them, angiotensin converting enzyme inhibitors (ACEIs) [2] and statins [126] seem to lower the incidence of POAF but evidence is still inconclusive. Conflicting results have also been reported for acetylcysteine [127], sodium nitroprusside [128], and glucocorticoids [129]. 

*(vi) Pacing*. Nonpharmacologic therapy with atrial pacing has been tested in various studies. The previously reported meta-analysis showed a significant reduction in AFib (OR 0.57, 95% CI 0.38–0.84) [71]. Most [130–132] but not all [133, 134] published studies showed benefit. Moreover, there are conflicting findings as to the relative value of the different types of pacing [130, 131].

A meta-analysis of 58 randomized trials of over 8,500 patients about the relative benefit from beta blockers, sotalol, amiodarone, and pacing was evaluated in a 2004 meta-analysis [71]. The incidence of AFib was reduced from 31–40% in controls to 18–22% with active therapy with the following odds ratios: 0.35 (95% CI 0.26 to 0.49) for beta blockers, 0.36 (95% CI 0.23 to 0.56) for sotalol, 0.54 (95% CI 0.44 to 0.67) for amiodarone, and 0.57 (95% CI 0.38–0.84) for pacing. Despite the significant reduction in AFib, prophylactic drug therapy was associated with a nonsignificant reduction in stroke (OR 0.76, 95% CI 0.43–1.31), which may have been due to a low rate of events (1.2% versus 1.4%) [71]. Another meta-analysis, including 94 trials of prophylactic therapy, found similar risk reductions with the four therapies noted above, as well as a benefit from magnesium [135]. This analysis also showed that the effect of beta blockers may have been overestimated in some studies.

## 4. Ventricular Tachyarrhythmias

### 4.1. Premature Ventricular Complexes

*(i) Isolated Premature Ventricular Complexes (PVC) Are Not Uncommon after Surgery*. PVCs can be related to electrolyte or other metabolic imbalances. PVCs are usually readily identified by surface ECG or continuous telemetric monitoring but sometimes must be distinguished from atrial ectopy with aberrant ventricular conduction. 

*(ii) Prognosis*. Patients with isolated and noncomplicated PVCs postoperatively do not exhibit increased risk of malignant ventricular arrhythmias [136, 137]. On the other hand, frequent PVCs (>30 per hour) may have an impact on short-term outcome by reducing ventricular function. A study [138] of 185 patients reported no significant differences in mortality rates at an average followup of 3 years in patients with versus patients without frequent postoperative PVCs and nonsustained ventricular tachycardia (NSVT) (8% versus 5%). However, another study [139] of 126 patients with postoperative complex ventricular ectopies showed that patients with impaired left ventricular ejection fraction (<40%) had a 75% mortality rate and 33% incidence of sudden death at an average followup of 15 months, whereas none of the patients with preserved left ventricular function had sudden death. Thus, long-term outcome after cardiac surgery seems to be closely related to the ventricular function. Ventricular arrhythmias occurring postoperatively could be considered in this view an epiphenomenon of ventricular systolic dysfunction. 

*(iii) Management*. Asymptomatic and hemodynamically stable PVCs do not usually need neither acute treatment nor long-term antiarrhythmic therapy. However, the correction of any reversible cause of ventricular arrhythmias should be pursued. Lidocaine has been used successfully in reducing hemodynamically significant or symptomatic PVCs, although without improving mortality. Several studies in other setting have shown that empirical suppression of frequent and/or complex PVCs with class I antiarrhythmic drugs had no beneficial effects on mortality rate and may be harmful [140, 141]. Also overdrive pacing, using either atrial or atrioventricular sequential pacing, has been used without significant results [140, 141]. Patients with preserved left ventricular ejection fraction and asymptomatic NSVT after cardiac surgery generally have a favorable long-term prognosis and do not require electrophysiology study. Implantable cardioverter defibrillators (ICDs) have shown no benefits in improving prognosis in this population [142].

### 4.2. Ventricular Tachyarrhythmias

*(i) Epidemiology*. Sustained ventricular arrhythmias are uncommon after surgery and include ventricular tachycardia (VT) and ventricular fibrillation (VFib). Reported incidences after cardiac surgery range from 0.41% to 1.4% [140, 143]. 

*(ii) Risk Factors*. Complex ventricular arrhythmias are associated with left ventricular dysfunction [139]. Other conditions associated with ventricular arrhythmias after cardiac surgery include hemodynamic instability, electrolyte imbalances, hypoxia, hypovolemia, myocardial ischemia and infarction, acute graft closure, reperfusion after cessation of cardiopulmonary bypass, and inotropes and antiarrhythmic drugs use. 

*(iii) Clinical Course and Diagnosis*. The hemodynamic state of patients with ventricular arrhythmias depends upon the rate of the tachyarrhythmia and left ventricular function. Based on ECG criteria, wide complex tachycardias may be either ventricular or supraventricular tachycardia. However, in patients with prior infarction, the diagnosis is mostly ventricular tachycardia. If feasible, a 12-lead electrocardiogram and atrial electrograms through temporary epicardial wires placed at the time of cardiac surgery should be obtained. The latter method would detect the presence of atrioventricular dissociation, which strongly suggests ventricular tachycardia [140]. 

*(iv) Prognosis*. The prognosis is correlated with the type of arrhythmia and the type and degree of structural heart disease. NSVT, as well as frequent PVCs, have generally no impact on outcome. Patients with sustained ventricular arrhythmias have poorer short- and long-term prognosis. An in-hospital mortality rate of up to 50% has been reported in patients with sustained ventricular arrhythmias after surgery. Among patients who survive in-hospital sustained ventricular arrhythmias, up to 40% have a recurrence. As many as 20% of these patients die from cardiac causes within 24 months [137, 138]. 

*(v) Therapy*. Asymptomatic and hemodynamically stable short runs of NSVT do not need acute treatment and the correction of any reversible cause of ventricular arrhythmias is generally sufficient. As previously stated, lidocaine and overdrive pacing have been used for hemodynamically significant or symptomatic episodes of NSVT, but evidence of their effectiveness is still controversial. 

Postoperative sustained ventricular arrhythmias treatment follows the indications used in other clinical settings [144]. However, the postoperative state requires a closer attention to the identification and treatment of electrolyte or other metabolic imbalances, myocardial ischemia, or mechanical complications of surgery. Sustained ventricular tachyarrhythmias should be promptly cardioverted either by drugs infusion or electrically. Hemodynamically stable sustained VTs may be initially treated with antiarrhythmic drugs intravenous infusion.

*(1) Lidocaine*. It is generally the first choice, even if there is not a complete consensus about its effectiveness. Lidocaine is given as a bolus of 0.75–1.5 mg/kg, followed by an intravenous continuous infusion of 1–4 mg/min. The maximal dosage is 3 mg/Kg/hour. The dosage should be reduced in patients who are elderly, have congestive heart failure, or have hepatic dysfunction [145]. 

*(2) Procainamide*. It is often the second line drug. It is given as a loading dose of 20–50 mg/min for a total dosage of 15 mg/kg, followed by an infusion at 1–4 mg/min. The loading doses should be stopped early if the QRS complex widens by >50% or if hypotension develops. Dosage modifications or even avoidance may be required in the presence of renal insufficiency. The N-acetyl-procainamide metabolite may accumulate to toxic levels in case of renal failure [145]. 

*(3) Amiodarone*. Intravenous amiodarone is frequently used as a first-line treatment for ventricular arrhythmias. A bolus of 300 mg is given over 1 hour, followed by infusion at 50 mg/hour. Additional 150 mg boluses may be given over the first few hours for recurrent hemodynamically significant VT/VFib. However frequent boluses during the first 24 hours should be limited for the risk of hepatic toxicity. Hypotension may require dose excalation. Amiodarone is often better tolerated in patients with systolic dysfunction than the other antiarrhythmic drugs [145]. 

*(4) Pacing*. In patients with slower ventricular tachycardias who are still retaining ventricular epicardial wires, overdrive pacing may be performed. Electrical cardioversion/defibrillation should be easily available, because acceleration of VT and degeneration to VFib are possible [146]. 

*(5) Electrical Cardioversion/Defibrillation*. Electrical defibrillation should be performed for VFib and hemodynamically unstable VT and electrical cardioversion for stable sustained VT either as the first choice or for those who do not respond to antiarrhythmic medications. Generally recommended energy levels with modern biphasic defibrillators range from 150 to 200 Joules. Sedation with short-acting agents should precede energy delivery in awake patients [146]. 

*(6) Emergency Measures*. Initiation of emergency cardiopulmonary bypass in the surgical intensive care unit can be considered for patients not responding to conventional resuscitation maneuvers. A 56% long-term survival rate for patients undergoing this procedure with no resultant mediastinitis and a 22% incidence of soft tissue infections has been reported [146]. 

*(7) Implantable Cardioverter-Defibrillator (ICD)*. An aggressive long-term management, potentially with electrophysiologic study and eventually ICD implantation, is often indicated, particularly in the absence of a reversible cause.

Patients with NSVT, prior myocardial infarction, and left ventricular dysfunction (left ventricular ejection fraction <40%) should be considered for electrophysiologic testing with implantation of an ICD if a sustained ventricular arrhythmia is induced. In the Multicenter Automatic Defibrillator Implantation Trial (MADIT), patients with coronary artery disease, left ventricular ejection fraction <35%, NSVT, and inducible sustained ventricular arrhythmias not suppressible by procainamide were randomized to prophylactic ICD implantation versus conventional medical therapy, mostly amiodarone [144]. A 54% reduction in mortality rate was reported in the ICD arm. In the Multicenter Unsustained Tachycardia Trial (MUSTT) [147], patients with coronary artery disease, left ventricular ejection fraction <40%, NSVT, and inducible sustained VT were randomized to electrophysiologic-guided therapy versus standard therapy (ACEIs and beta blockers). There was a 27% reduction in arrhythmic death and cardiac arrest in the electrophysiologic-guided group compared with conventional treatment group. All of this risk reduction was seen in patients that received an ICD. However, applicability of these studies to NSVT occurring in the immediate postcardiac surgery setting is not well established. The Coronary Artery Bypass Graft Patch Trial (CABG-Patch) [148] showed no mortality differences among patients with low ejection fraction and a positive signal-averaged electrocardiogram randomized to ICD or no ICD implantation at the time of CABG surgery. In this setting CABG surgery may reduce arrhythmic risk by relief of ischemia-provoked arrhythmias. Patients who underwent CABG surgery within 2 months were excluded from the MADIT trial. The MUSTT trial, however, did include patients who had NSVT four or more days after revascularization. The postoperative monitoring period often permits the detection of this type of arrhythmias, especially in patients with ischemic cardiomyopathy. Thus, detection of several episodes of NSVT in this setting warrants risk stratification as suggested by the MADIT and the MUSTT trials. Empirical amiodarone showed no improvement in overall mortality rates (despite a reduction in arrhythmic deaths) in patients with ischemic cardiomyopathy in the European Myocardial Infarct Amiodarone Trial (EMIAT) [149] and Canadian Amiodarone Myocardial Infarction Arrhythmia Trial (CAMIAT) [150]. The EMIAT excluded patients waiting for cardiac surgery. Management of patients with nonischemic cardiomyopathy and NSVT is even less clear. The Grupo de Estudio de la Sobrevida en la Insuficiencia Cardiaca en Argentina (GESICA) study [151] suggested an improvement in mortality rate with empirical amiodarone in heart failure patients, but the Congestive Heart Failure Survival Trial of Antiarrhythmic Therapy (CHF-STAT) [152] showed no benefit with the same strategy. The GESICA study had a higher proportion of patients affected by nonischemic cardiomyopathy, suggesting a possible benefit from prophylactic amiodarone in this population. The CHF-STAT study excluded patients within 3 months of revascularization. In patients with sustained ventricular arrhythmias the implantation of an ICD is recommended as a first-line therapy because recurrence rate and long-term mortality rate are high. The Antiarrhythmics Versus Implantable Defibrillators (AVID) trial [153] and other trials have demonstrated the effectiveness of ICD over drug therapy in improving prognosis in secondary prevention of patients who showed hemodynamically significant ventricular arrhythmias. Although the applicability of these results to sustained ventricular arrhythmias in the immediate postoperative period is not established, in the AVID trial about 10% of patients in the ICD group and 12% of patients in the antiarrhythmic drug group underwent coronary revascularization during hospitalization for the index arrhythmia. 

*(8) Standard Medical Therapy*. Other drugs have been demonstrated to improve long-term survival. These drugs are particularly recommended in patients with left ventricular dysfunction. These include beta blockers and ACEIs. Moreover statins and polyunsaturated free fatty acid have been demonstrated to reduce overall and cardiac deaths [145].

## 5. Bradyarrhythmias

### 5.1. Epidemiology and Risk Factors

Bradyarrhythmias are common after cardiac surgery. In the majority of cases they consist of transitory episodes of low ventricular heart rate. They usually include sick sinus syndrome (SSS) and various degree of atrioventricular blocks (AVB). Bradyarrhythmias may decrease cardiac output in patients with relatively fixed stroke volumes.

Bradyarrhythmias are particularly common after valve surgery and are a consequence of direct surgical injury and local edema. After valve surgery and CABG bradycardia usually is caused by complete or high-degree AVB and requires permanent pacing in 2% to 4% of patients [154, 155]. After CABG surgery, permanent pacing is required for sinus node dysfunction or atrioventricular conduction disturbances in 0.8% to 3.4% of patients. Permanent pacing may be required in up to 20% to 24% after some types of procedures, such as for calcific aortic stenosis or tricuspid valve replacement. Significant risk factors for high-degree AVBs include perivalvular calcification, older age, preoperative left bundle branch block, left ventricular aneurysmectomy, left main coronary artery stenosis, number of bypassed arteries, and cardiopulmonary bypass time. Mitral valve repair and replacement have been associated with similar frequencies of second- or third-degree AVB with new conduction disturbances occurring in 30.6% of patients and complete heart block in 1.5% of patients [155]. The right lateral atriotomy used in minimally invasive mitral valve operations or other transseptal superior approaches to the mitral valve can cause SSS with persistent symptomatic sinus bradycardia or junctional rhythms requiring permanent pacing. Permanent pacemakers are required more often after repeat valve surgeries (7.7% versus 2.0% after initial valve surgery) [154]. A complete heart block might also develop following an AFib catheter or surgical ablation procedure, especially when radiofrequency energy is delivered nearby the septal region [156]. After orthotopic heart transplantation, SSS is common and leads to permanent pacemaker implantation in up to 21.1% (mean, 8%) of patients. AVB is less common but requires pacemaker implantation in up to 4.5% of transplant recipients. Permanent pacing is needed less frequently after bicaval compared with biatrial transplant anastomosis [157]. Bradyarrhythmias from acute or chronic rejection can also lead to permanent pacing. Predictive factors for bradyarrhythmias after heart transplantation include older donor age, longer donor ischemic time, and longer aortic cross-clamp time. Sinus node function after orthotopic heart transplantation often improves over long-term followup, but recovery might proceed over weeks to months, commonly hampering expeditious hospital discharge. The need for long-term pacing may be unpredictable, although patients with early AVB may be more likely to require it. Chronotropic medications, such as theophylline or aminophylline, have been used for sinus bradycardia after transplantation to improve SSS [158] or high grade AVB [159] and may decrease the need for permanent pacing.

### 5.2. Management

Temporary electrical pacing may be required in symptomatic bradycardias. In some cases, when the conduction defect does not revert, permanent pacing may be necessary. Temporary epicardial atrial and ventricular pacing wires placed at the time of surgery usually facilitate temporary pacing.

The frequent challenge with postoperative bradycardia is often to determine how long to wait to allow recovery of sinus node function or atrioventricular conduction after surgery before implantation of a permanent pacemaker. Recovery is common with long-term followup. Among patients who receive permanent pacing, only 30% to 40% of patients with SSS remain pacemaker dependent. The rate of recovery is less with AVB. Among patients with complete heart block, 65% to 100% remain dependent. A usual practice is to implant a permanent pacemaker if symptomatic complete AVB or severe SSS persists longer than 5–7 days postoperatively [160]. If underlying intrinsic rhythm is absent or temporary pacing leads fail, permanent pacing may be performed earlier.

## 6. Conclusion

Postoperative arrhythmias are frequent in the cardiac surgery setting. The most frequently observed POAs are supraventricular tachyarrhythmias, especially AFib. The general issues for the treatment of supraventricular arrhythmias are similar to those recommended in other settings. The treatment of postoperative ventricular arrhythmias is less clear but is similarly based on general indications for the treatment of ventricular arrhythmias. Finally, bradyarrhythmias are also frequently observed after cardiac surgery due to the conduction system trauma. Although conduction disturbances often recover spontaneously, permanent pacemaker implantation may be required.

## Abbreviations

ACEI: Angiotensin converting enzyme inhibitor
AFib: Atrial fibrillation
AFlu: Atrial flutter
ARB: Angiotensin II receptor blocker
AVB: Atrioventricular block
CABG: Coronary artery bypass graft
CCB: Calcium channel blocker
ICD: Implantable cardioverter-defibrillator
NSVT: Nonsustained ventricular tachycardia
POA: Postoperative arrhythmia
POAF: Postoperative atrial fibrillation
PVC: Premature ventricular complex
SSS: Sick sinus syndrome
VFib: Ventricular fibrillation
VT: Ventricular tachycardia.
